# Genome assembly and transcriptomic analyses of the repeatedly rejuvenating jellyfish *Turritopsis dohrnii*

**DOI:** 10.1093/dnares/dsac047

**Published:** 2022-12-15

**Authors:** Yoshinori Hasegawa, Takashi Watanabe, Reo Otsuka, Shigenobu Toné, Shin Kubota, Hideki Hirakawa

**Affiliations:** Department of Applied Genomics, Kazusa DNA Research Institute, Kisarazu, Chiba 292-0818, Japan; Department of Applied Genomics, Kazusa DNA Research Institute, Kisarazu, Chiba 292-0818, Japan; Graduate School of Science and Engineering, Tokyo Denki University, Hatoyama, Saitama 350-0394, Japan; Graduate School of Science and Engineering, Tokyo Denki University, Hatoyama, Saitama 350-0394, Japan; Turritopsis Immortal Jellyfish Regenerative Biological Research/Experience Laboratory, Arita, Wakayama 643-0002, Japan; Department of Applied Genomics, Kazusa DNA Research Institute, Kisarazu, Chiba 292-0818, Japan

**Keywords:** *Turritopsis*, jellyfish, rejuvenation, genome assembly, mRNA-Seq

## Abstract

Only two hydromedusan species, *Turritopsis dohrnii* and *T*. sp., have exhibited experimental multiple-repeat life cycle reversion in the laboratory, which can be artificially induced by various means such as incubation with CsCl, heat shock, and mechanical damage with needles. In the present study, we constructed a genome assembly of *T. dohrnii* using Pacific Biosciences long-reads and Illumina short-reads, for which the genome DNA was extracted from 1,500 young medusae originated from a single clone. The total length of the draft genome sequence of *T. dohrnii* was 435.9 Mb (N50 length 747.2 kb). We identified 23,314 high-confidence genes and found the characteristics of RNA expression amongst developmental stages. Our genome assembly and transcriptome data provide a key model system resource that will be useful for understanding cyclical rejuvenation.

## 1. Introduction

Unlike almost all multicellular animals, including most jellyfish species, some cnidarian species show life cycle reversion,^1–8^ in which mature and young medusae transform directly into an earlier developmental stage namely, the polyp stage (Supplementary Fig. S1). Amongst such species, *Turritopsis dohrnii* has been artificially rejuvenated by many means, such as incubation with CsCl and heat shock.^5^ Moreover, *Turritopsis* sp. showed as many as 10 repeated cycles of rejuvenation in response to severe mechanical damage with needles.^7^ In these two species, *de novo* RNA-Seq analyses were performed to uncover clues about the process of rejuvenation.^9–11^ Although the results provided valuable information about the characteristics of rejuvenation, whole genome sequence analysis is necessary for a detailed interpretation of rejuvenation mechanisms. The genome sequences of diversity of jellyfish have been reported, including the same class of hydrozoa, *Aurelia* sp.1 (Scyphozoa),^12^*Clytia hemisphaerica* (Hydrozoa),^13^*Alatina alata* (Cubozoa),^14^*Calvadosia cruxmelitensis* (Staurozoa),^14^*Cassiopea xamachana* (Scyphozoa),^14^*Nemopilema nomurai* (Scyphozoa),^15^*Morbakka virulenta* (Cubozoa),^16^*Aurelia aurita* (Scyphozoa),^16^*Rhopilema esculentum* (Scyphozoa),^17,18^*Sanderia malayensis* (Scyphozoa),^18^ and *Chrysaora quinquecirrha* (Scyphozoa).^19^ However, the genome sequences of *Turritopsis* species by long-reads have not been determined, although the genome assembly of *T. dohrnii* using only Illumina short reads was reported recently.^20^ The body size of mature medusae of *T. dohrnii* from Wakayama prefecture, Japan, is about several millimetres. From the point of view of whole genome analysis, it is desirable to prepare microgram-order DNA from a single individual. However, it is impossible to extract a sufficient amount of DNA from a single animal with such a tiny body. Recently, our group succeeded in the clonal breeding of *T. dohrnii*,^21^ and we managed to collect a total of 3 µg genomic DNA using over 1,500 young medusae that originated from a single clone. In this study, we have determined the draft genome sequence of *T. dohrnii* by using the long-read sequencer, Sequel (Pacific Biosciences, CA). The genes were predicted on the draft genome sequence and compared to the genes of the other cnidarians to estimate a phylogenetic relationship. Furthermore, we searched the conserved genes amongst the four species, *T. dohrnii*, *Aurelia aurita*, *Clytia hemisphaerica*, and *Hydra magnipapillata*, and finally conducted a functional characterization of the unique genes found in each species.

## 2. Materials and methods

### 2.1. Sample collection for DNA, genomic DNA extraction, and sequel or illumina library preparation

A *Turritopsis* medusa was collected on 17 August 2017, in Tanabe Bay, Wakayama, Japan (33.68ʹE, 135.36ʹN). This medusa was confirmed as *T. dohrnii* by its 16S ribosomal DNA sequence.^22^ It is noted that *T. dohrnii* and *T.* sp2 are sympatrically distributed in Tanabe Bay and cannot be distinguished based on morphological differences, but there are clear differences in rDNA sequences between the two species.^22,23^ We have established a breeding system using artificial seawater prepared with sea salt powder (Livesea Salt; Delphis, Hyogo, Japan) at 40 g per 1 l of milliQ water and filtered with paper filters. Its salinity was measured with a Marine Salt Testa metre (Marfied, Singapore) at 28 ppt. Polyps were maintained on plastic dishes in a water-circulating tank at 25°C. ^21^ Each polyp consists of all zooids (individual uprights from the stolon) in a colony, and each zooid consists of hydrocaulus, hydranth, and stolon. One simple unit of a colonial polyp is a zooid. The zooids were fed daily with hatched brine shrimps (Japan Pet Design, Tokyo, Japan). Then, 3 µg of DNA was extracted from about 1,500 young medusae of a colony, which was named the TD1 (Tokyo Denki University 1st) colony, using a Wizard Genomic DNA Purification Kit (Promega, Madison, WI). For the *de novo* assembly of *T. dohrnii* genome, PacBio SMRT libraries with 15 kb peak insert sizes were constructed according to the manufacturers’ protocols. A 20 ng aliquot of DNA from the total volume of 3 µg DNA was used for Illumina library preparation using a NEBNext Ultra II FS DNA Library Prep Kit for Illumina (New England Biolabs, Ipswich, MA), and sequenced on a NextSeq 500 system by performing 151 bp paired-end (PE) reads.

### 2.2. Sample collection for RNA, total RNA extraction, mRNA-Seq library preparation

To collect the largest possible amount of various mRNAs of *T. dohrnii*, we sampled the mRNAs from nine different developmental stages. After unbranched short zooids were fasted for 3 days, a total of 34 samples were collected from the nine developmental stages were collected at 0 h, 3 h, 6 h, 9 h, 16 h, 20 h, 24 h, 33 h, and 51 h-d (dumpling) or 51 h-s (stolon). The period of stages 0–9 h covered dumpling formation; that of 16–24 h covered the period up to the start of stolon budding; and the period from 33–51 h covered stolon elongation and the division of stolon and dumpling (Supplementary Fig. S1). All cut zooids were bred in a 6 cm plastic dish in an incubator at 25°C. The 0 h samples were frozen in liquid nitrogen immediately after cutting. At 51 h after cutting, the stolon was grown up from the dumpling, so the stolon and dumpling were divided. All stages were analysed with 3 or 4 biological replicates. The total RNA of each sample was extracted after immersion in 500 µl of Trizol (Life Technologies, Carlsbad, CA). After the addition of 100 µl chloroform followed by centrifugation, the aqueous phase was carefully transferred to a new tube, after which 20 µg of glycogen (Life Technologies) was added to each sample as a co-precipitant. RNA was precipitated by adding 250 µl of isopropyl alcohol. The RNA pellet was washed once with 70% ethanol and then dissolved in 12 µl RNase-free water. The quality and concentration of the RNA were verified with the Agilent 2100 Bioanalyzer and Qubit Fluorometer (Thermo Fisher Scientific, Waltham, MA), respectively. The final amount of each sample ranged from 3 to 40 ng RNA. For mRNA-Seq, 1 ng of purified total RNA was used for library preparation, according to the instructions of the NEBNext Ultra RNA Library Prep Kit for Illumina (New England Biolabs). The RNA libraries were sequenced on an Illumina HiSeq 2500 system with 100-bp PE reads for mRNA-Seq. The raw data were deposited to the DNA Data Bank of Japan (DRA BioProject accession number PRJDB11116). The adapter sequences were removed from the raw reads by using a FASTX toolkit v0.0.13. Quality trimming was performed for the 3ʹ end of each read to remove bases with quality below Q30 up to a minimum length of 25 bp by using PRINSEQ v0.20.4. Reads shorter than 25 bp were removed prior to further analysis.

### 2.3. *De novo* genome assembly, gene prediction, and gene annotation

The genome size of *T. dohrnii* line TD-1 was estimated by a peak position of the *k*-mer frequency plot obtained by Jellyfish v2.2.6^24^ using the Illumina PE reads. The PacBio long-reads were assembled using FALCON v2017.11.02-16.04 with the following parameters,^25^ length cutoff: −1, seed coverage: 100, pa_daligner_option: −e0.75 -k16 (parameter set A in Supplementary Table S3), and the resultant primary contigs and associated contigs were applied to FALCON-Unzip for phasing.^25^ The primary contigs and haplotigs were polished with the long-reads and Illumina PE short-reads by Arrow (https://github.com/PacificBiosciences/gcpp) and Pilon v1.23,^26^ respectively. The haplotic duplication in primary contigs was removed by Purge_haplotigs v1.1.1.^27^ The genes were predicted by BRAKER2 v2.1.5^28^ using mRNA-Seq data obtained from the nine stages (0 h, 3 h, 6 h, 9 h, 16 h, 20 h, 24 h, 33 h, 51 h) in this study. The gene with the highest score was selected from the predicted splicing variants in each gene locus. The selected genes were annotated by similarity searches against the UniProtKB database (https://www.uniprot.org) with *E*-value ≤ 1e−10 and NCBI’s NR database (ftp://ftp.ncbi.nlm.nih.gov/blast/db/FASTA/) with *E*-value ≤ 1e−10 by DIAMOND v0.9.29.130 in the more-sensitive mode.^29^ The genes were searched against the protein sequences of *Aurelia aurita* AUR (PRJNA494057) and *A. aurita* complex sp. Pacific (ARS; PRJNA494062) released from the Okinawa Institute of Science and Technology Graduate University (OIST),^16^ and of *Clytia hemisphaerica*^13^, *Hydra magnipapillata*^30^ by BLAST+ with *E*-value ≤ 1e−10. The genes were also searched against the protein domain database Pfam v32.0^31^ with *E*-value ≤ 1.0 by HMMER v3.2.1.^32^ The mRNA-Seq reads were mapped on the predicted genes by Salmon v1.2.1^33^ to calculate the expression value in transcripts per million (TPM). The predicted genes were categorized into three groups, namely high confidence (HC), low confidence (LC), and transposable element (TE). The HC category included the genes having hits against UniProtKB, NR, and the proteins of *A. aurita* released from OIST, *C. hemisphaerica*, *H. magnipapillata*, and against the protein domains of Pfam, and those with TPM values > 0.0 were considered to be expressed. The LC category contained the genes with a 0.0 TPM value and those with hits or no hits against the databases described above. The genes having hits against UniProtKB with keywords related to TEs were categorized into the TE category. The protein sequences of the HC genes of *T. dohrnii* line TD-1 were considered as intrinsic genes and used for further analyses. The predicted genes were compared with those of *A. aurita*, *C. hemisphaerica*, and *H. magnipapillata* by OrthoFinder v2.3.11.^34^ The gene conservation amongst the four species was represented on a Venn diagram. The genes were searched against the EggNOG database^35^ using eggnog-mapper v2.1.6^36^. The characteristics of the genes classified into each region were investigated by GO enrichment analysis by using TopGO (https://bioconductor.org/packages/release/bioc/html/topGO.html) against the results of the eggnog-mapper.

### 2.4. Repetitive sequences

Repetitive sequences were *de novo* detected by RepeatModeler v1.0.11 (http://www.repeatmasker.org/RepeatModeler/). The repetitive sequences were searched against the assembled genome sequence using the repetitive sequences found by RepeatModeler and Repbase^37^ with RepeatMasker v4.0.7 (http://www.repeatmasker.org). The repetitive sequences detected in the assembled genome sequence by RepeatModeler and RepeatMasker were softmasked using RepeatMasker.

## 3. Results and discussion

### 3.1. Genome sequencing

**Figure 1.**
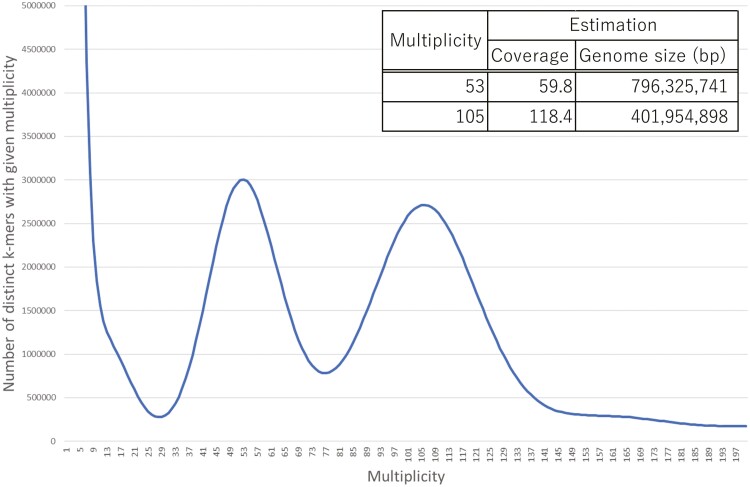
*k*-mer frequency plot of the paired-end reads. The plot was written for the *k*-mer frequencies calculated by Jellyfish using *k*-mer size 17. The genome size was estimated 401,954,898 bp.

We obtained approximately 3 µg DNA from over 1,500 young medusae. The statistics of the long-read sequences for 7 SMRT cells obtained by Sequel are summarized in Supplementary Table S1. The total length of the reads was 88,253,394,018 bp, with an average N50 length of 9175 bp. The statistics of PE short-read sequences for four lanes are also summarized in Supplementary Table S1. The total length of the reads was 47,583,881,671 bp, with an average length of 151 bp. The *k*-mer frequency plot of the PE reads is shown in Fig. 1. The genome size of *T. dohrnii* line TD-1 was estimated to be 401,954,898 bp based on the *k*-mer frequency plot. Based on the amount of reads, the coverages of the genomic long-reads and short-reads were 219.6 and 118.4, respectively.

### 3.2. mRNA sequencing

The mRNA was sequenced from the samples at 0 h (4 samples), 3 h (4 samples), 6 h (4 samples), 9 h (4 samples), 16 h (3 samples), 20 h (3 samples), 24 h (3 samples), 33 h (3 samples), and 51 h (3 samples from c and 3 from s) using HiSeq 2500. The statistics of the short-read sequences for mRNA are summarized in Supplementary Table S2. In total, 2,250,702,988 mRNA-Seq reads with 225.9 Gb were obtained.

### 3.3. *De novo* genome assembly

**Figure 2.**
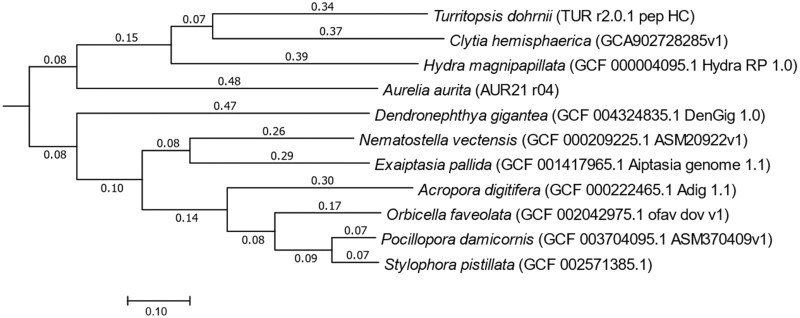
Phylongenetic relationship amongst the 11 metazoan species. The dendrogram was constructed based on the orthogroups calculated by OrthoFinder. The distances were shown on each branch. The NCBI accession numbers of the genomes are as follows, *Pocillopora damicornis* (GCF_003704095.1_ASM370409v1), *Stylophora pistillata* (GCF_002571385.1_Stylophora_pistillata_v1), *Orbicella faveolata* (GCF_002042975.1_ofav_dov_v1), *Acropora digitifera* (GCF_000222465.1_Adig_1.1), *Nematostella vectensis* (GCF_000209225.1_ASM20922v1), *Exaiptasia pallida* (GCF_001417965.1_Aiptasia_genome_1.1), *Dendronephthya gigantea* (GCF_004324835.1_DenGig_1.0), *Hydra magnipapillata* (GCF_000004095.1_Hydra_RP_1.0), and *Clytia hemisphaerica* (gca902728285.GCA902728285v1). The genomic data of *Aureria aurita* (AUR21_r04) were downloaded from the download site at OIST Marine Genomics Unit under https://marinegenomics.oist.jp/aurelia_aurita/viewer/download?project_id=69). Phylogenetic relationships amongst them were inferred by Species Tree Inference from All Genes (STAG) method (bioRxiv;https://www.biorxiv.org/content/10.1101/267914v1) using OrthoFinder. The phylogenetic tree was constructed by using MEGA 11 v11.0.11.

A total of 88.3 Gb of subread sequences generated by 7 SMRT cells of Sequel (Supplementary Table S1) were subjected to *de novo* assembly by FALCON v 1.4.2, and 1843 primary contigs (total length: 542.8 Mb; N50 length: 641.5 kb) and 1068 associate contigs (total length: 80.0 Mb; N50 length: 130.4 kb) were constructed (Supplementary Table S3). The primary and associate contigs were phased by FALCON-Unzip v1.3.3, and 1448 primary contigs (total length: 529.9 Mb; N50 length: 639.3 kb) and 2020 haplotigs (total length: 137.7 Mb; N50 length: 160.7 kb) were constructed (Supplementary Table S3). The haplotic duplication in primary contigs was removed by Purge_haplotigs with 65% cutoff for the alignment score to identify haplotigs. The resultant genome sequence was designated TUR_r2.0. The contig ‘Tur_r2.0_p0000.1’ was excluded due to probable contamination from TUR_r2.0. Finally, 891 primary contigs with a total length of 435.9 Mb (N50 length: 747.2 kb) were constructed and were designated TUR_r2.0.1 (Table 1) (GenBank accession numbers: BQMF01000001-BQMF01000891). The heterozygosity was 1.88 calculated by GenomeScope with *k*-mer size 17. The BUSCO completeness using metazoa_odb9 of TUR_r2.0.1 was 90.4%, which was higher than those of the other cnidarian genomes (Supplementary Table S3). Phylogenomic analysis based on orthogroups together with 10 cnidarian species showed that two main clades were generated. The clade including *T. dohrnii* consisted of four species, two Hydrozoa species, which is the same class as *T. dohrnii*, *C. hemisphaerica*, *H. magnipapillata,* and one Scyhphozoa species *A. aurita* (Fig. 2).

**Table 1.** Statistics of the genome sequence TUR_r2.0.1 of *Turritopsis dohrnii* line TD-1

		TUR_r2.0.1
Total	Number of sequences	891
	Total length (bases)	435,923,997
	Average length (bases)	489,253
	Max length (bases)	3,379,503
	Min length (bases)	20,407
	N50 length (bases)	747,194
	A	142,378,549
	T	142,396,835
	G	75,558,372
	C	75,590,241
	GC% (ATGC)	34.7
BUSCO odb9 (Metazoa; 978)	Complete%	90.4
	Complete and single	81.4
	Complete and duplicated	9.0
	Fragmented%	1.6
	Missing%	8.0

### 3.4. Repetitive sequences

The repetitive sequences detected in TUR_r2.0.1 and the other cnidarian species, *A. aurita*, *C. hemisphaerica*, and *H. magnipapillata*, are summarized in Supplementary Table S4. The percentages of the known repetitive sequences in TUR_r2.0.1, *A. aurita*, and *C. hemisphaerica* were 21.0%, 13.2%, and 16.0%, respectively, whereas that in *H. magnipapillata* was 53.8%. The total length of *H. magnipapillata* (852,170,992 bp) was greater than those of TUR_r2.0.1 (435,923,997 bp) (Supplementary Table S3). The Class II DNA elements of *H. magnipapillata* accounted for 30.3% of the total, which was a larger portion than those of TUR_r2.0.1 (9.4%), *A. aurita* (4.0%), and *C. hemisphaerica* (6.5%). On the other hand, the unique repeats found in TUR_r2.0.1, *A. aurita*, and *C. hemisphaerica* accounted for 39.3%, 32.1%, and 33.9% of the total, respectively, whereas those in *H. magnipapillata* accounted for 8.5%. In total, the percentages of the known and unique repeats were not largely different amongst the four species (TUR_r2.0.1 (60.4%), *A. aurita* (66.6%), *C. hemisphaerica* (49.9%), and *H. magnipapillata* (62.3%)).

### 3.5. Gene prediction and annotation

A total of 75,313 genes were initially predicted from the softmasked TUR_r2.0.1 sequences by BRAKER2 using mRNA-Seq data sampled from the 9 stages (0 h, 3 h, 6 h, 9 h, 16 h, 20 h, 24 h, 33 h, 51 h) in this study. Of those, 73,694 genes (hereafter referred to as the “best” genes) having the highest scores amongst the splicing variants were selected (Supplementary Table S5). The ‘best’ genes were subjected to DIAMOND searches in the more-sensitive mode against the UniProtKB and NCBI NR databases, BLAST+ searches against the protein sequences of *A. aurelia* AUT and ARS, and domain searches against the Pfam 32.0 database. Then, 26,222 genes with TPM values >0 calculated by Salmon were considered to be expressed. The ‘best’ genes were classified into the HC (high-confidence), LC (low-confidence), and TE (transposable element) categories according to the similarity searches and expression values described above and the categorized gene sets were named TUR_r2.0.1 after removal of the contaminated genome sequence ‘Tur_r2.0_p0000.1’. The numbers of genes classified into HC, LC, and TE were 23,314, 34,177, and 12,427, respectively (Supplementary Table S5). The HC genes of TUR_r2.0.1 were found to have high completeness, with a BUSCO score of 92.4%, whereas the LC and TE genes of TUR_r2.0.1 showed extremely low BUSCO completeness scores of 5.5% and 1.3%, respectively. These results suggest that the intrinsic genes were correctly selected as high-confidence (HC) genes from the ‘best’ genes predicted by BRAKER2. The genes related to TEs were also selected from the ‘best’ genes. The remaining genes were classified as LC genes, and most of them were unlikely to be intrinsic to *T. dohrnii*. Nonetheless, the LC genes can be an informative sequence set to search for novel functional genes, particularly when combined with the expression evidence obtained from future RNA-seq studies of as-yet-unexamined organs/tissues. The statistics of the intrinsic genes predicted as HC are summarized in Table 2. The genome and predicted genes are publicly available in the *Turritopsis* Genome DataBase (http://turritopsis.kazusa.or.jp).

**Table 2.** Statistics of the genes predicted from TUR_r2.0.1

		TUR_r2.0.1 (HC)
Total	Number of sequences	23,314
	Total length (bases)	38,406,405
	Average length (bases)	1,647
	Max length (bases)	55,572
	Min length (bases)	126
	N50 length (bases)	2,250
	A	12,740,404
	T	10,519,010
	G	8,105,990
	C	7,040,886
	N	115
	GC% (ATGC)	39.4
BUSCO odb9 (Metazoa; 978)	Complete%	92.4
	Complete and single	82.7
	Complete and duplicated	9.7
	Fragmented%	3.0
	Missing%	4.6

### 3.6. Gene comparison amongst the other cnidarian species

Supplementary Table S5 summarizes the status of the genes in *Turritopsis dohrnii* line TD-1 and the genes of the three cnidarian species. As described above, the BUSCO completeness scores of the HC genes of TUR_r2.0.1 were high, at 92.4%, which was higher than those of *A. aurita* (BUSCO completeness%: 82.9%), *C. hemisphaerica* (84.3%), and *H. magnipapillata* (91.5%). The peptide sequence similarities of the genes from the two jellyfish species and another cnidarian species, *Hydra*, were compared by OrthoFinder v2.5.4. The distribution of the genes amongst the four species is summarized by a Venn diagram (Supplementary Fig. S2). Commonly distributed amongst the four species were 6,297 orthogroups. In the intersection of the four species, 8,539, 11,501, 9,522, and 9,382 genes were respectively included in *T. dohrnii*, *A. aurita*, *C. hemisphaerica*, and *H. magnipapillata*. There were 1,119, 3,058, 1,647, and 803 orthogroups respectively found in *T. dohrnii*, *A. aurita*, *C. hemisphaerica*, and *H. magnipapillata*, and these orthogroups respectively included 5,377, 13,457, 6,927, and 4,994 genes. Amongst them, the numbers of unassigned genes having no hits against the other species were 1,883, 6,083, 2,757, and 1,886, respectively. The functional categories of the genes were investigated by eggnog-mapper. The annotation performed by eggnog-mapper and DIAMOND search against UniProtKB for the 7,260 genes assigned into the orthogroups (1,883 genes) and the unassigned genes (5,377 genes) in TUR_r2.0.1 is summarized in Supplementary Table S6. In the result of eggnog-mapper, the NCBI’s COG/KOG categories, descriptions of the gene products, gene IDs, GO IDs, EC numbers, KEGG IDs (IDs for KO, Pathway, Module, Reaction, Rclass), KEGG BRITE, KEGG TCs, BiGG Reaction, and PFAMs are summarized. According to the hits against UniProtKB and EggNOG, the genes that may be related to the characteristics of the *T. dohrnii* were appeared. In the UniProtKB search, there are 146 ‘ATP-dependent DNA helicases’, one ‘chromodomain-helicase-DNA-binding-protein 1-like’, one ‘DNA helicase’, two ‘caspase-3-like’, two ‘programmed cell death protein6-like’, and one ‘nuclear apoptosis-including factor 1’. According to the GO IDs assigned by EggNOG search, GO enrichment analysis was conducted for the genes in the Venn diagram region at the intersection of the four species. The GO categories in BP (Biological Process), CC (Cellular Component), and MF (Molecular Function) with the top 20 lowest *p*-values are summarized in Supplementary Table S7. The gene ontologies found with especially low *p*-value (*p* < 1e−30) were found in *T. dohrnii* (CC; GO:0005887: integral component of the plasma membrane, GO:0031226: intrinsic component of plasma membrane) and *C. hemisphaerica* (CC; GO:0005576: extracellular region, GO:0071944: cell periphery, GO:0005615: extracellular space, GO:0030312: external encapsulating structure, MF; GO:0031996: thioesterase binding). The function of the genes described above should be investigated for our further studies.

### 3.7. mRNA-Seq analyses amongst rejuvenation-stages

The RIN value of the extracted total RNA of all samples was >7. Illumina sequencing of the 34 libraries from the 9 developmental stages yielded 2.25 billion 100-bp paired-end reads of mRNA-Seq (Supplementary Table S2). Each read of the stage-specific library was mapped to the draft genome sequence TUR_r2.0.1 using HISAT2 v2.2.1.^38^ The mapping percentages of all stages are summarized in Supplementary Table S8. Hereafter, all RNA-Seq analyses were conducted using only 23,314 HC genes.

**Figure 3.**
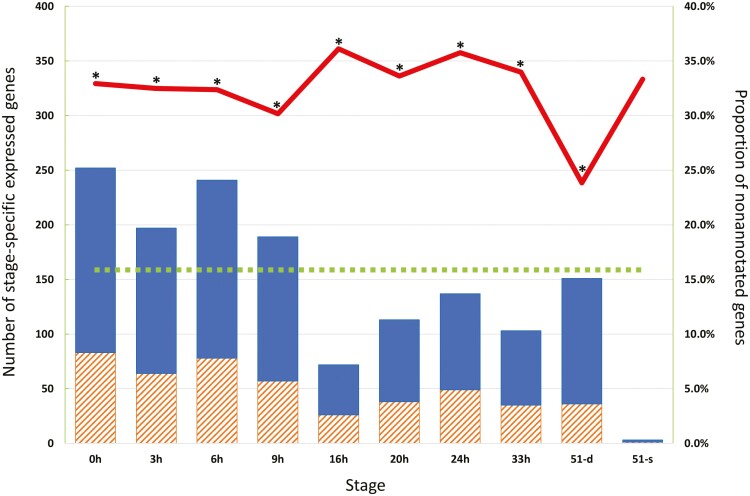
Proportion of nonannotated genes in each stage-specific expression gene in each of the nine developmental stages. Slated orange and blue solid bars showed no-hit and hit genes in UniProtKB analysis, respectively. The Red line shows the proportions of no-hit genes in UniProtKB analysis. The green dotted line shows no-hit genes in UniProtKB analysis amongst all 23,314 HC genes, 15.9%. The asterisk shows a significantly different proportion (*p* < 0.01) between the red line and the green dotted line.

The similarity searches against UniProtKB by DIAMOND and the expression (TPM) values in each sample are shown in Supplementary Table S9. The no-hit genes against UniProtKB, which are possibly *T. dohrnii*-specific genes, accounted for 15.9% of the 23,314 HC genes, whereas 23.8–36.1% of all developmental stage-specific expression genes, whose TPMs were zero at all other stages, were no hits (Fig. 3). On the other hand, few HC genes hit to the human pluripotent stem cell markers including iPS Yamanaka factors,^39^ such as LIN28, SSEA1, KLF4, OCT3, SOX2, and NANOG. These results suggest that the no-hit genes in the UniProtKB analysis could contribute significantly to the dedifferentiation and redifferentiation cycle of *Turritopsis* during rejuvenation and regeneration, and that dedifferentiation of *Turritopsis* might be controlled by different mechanisms of human. The summary table of the gene annotation by DIAMOND searches against UniProtKB, expression values (TPM) calculated with Salmon, eggnog-mapper search against EggNOG, and BLAST searches against the protein sequences of *Aurelia aurita* (AUR), and TPM values in each sample were published from the *Turritopsis* Genome DataBase as Supplementary Table S10.

## 4. Conclusion and future perspectives

We have sequenced and assembled the genome of *T. dohrnii*. The quality of the assembled genome compares favourably with those of cnidarian species reported previously (Supplementary Table S3). The total length of the genome sequence of *T. dohrnii* was 435,923,997 bp, however, the estimated genome size using the peaks in the *k*-mer frequency plot was 401,954,898 bp. In 2022, the genome sequence of *T. dohrnii* S-001 collected in Santa Caterina, Lecce has been first published in the genus *Turritopsis*.^20^ They estimated that the genome size of *T. dohrnii* was approximately 390 Mb. The total and N50 lengths of the genome sequence are 400.1 Mb and 10.4 kb, respectively (BUSCO completeness: 82.6%; NCBI BioProject: PRJNA734867). The genome size estimated in our study (402.0 Mb) corresponds well to the assembly size of the *T. dohrnii* S-001 genome (400.1 Mb). The number of the genes predicted was 17,469 (BUSCO completeness: 53.8%) in *T. dohrnii* S-001 (Supplementary Table S5). Besides, we have assembled the genome sequences by using another assembler to evaluate the genome size of *T. dohrnii* TD-1. We applied the FALCON with two parameter sets, Canu v1.7.1^40^, Wtdbg2 v2.4^41^, Flye v2.9^42^, and NextDenovo (https://github.com/Nextomics/NextDenovo). In the case of NextDenovo, we further excluded duplicated haplotigs by Purge_haplotigs. The assembly results were summarized in Supplementary Table S3. In the result of FALCON with parameter sets B and C (B; length_cutoff: −1, seed coverage: 100, pa_daligner_option: −e0.8 −k18, C; length_cutoff: 5000, pa_daligner_option: −e0.8 −k18), total lengths were 498.5 Mb and 498.7 Mb with N50 length 362.5 kb and 361.1 kb, respectively. In the result of FALCON with parameter set A described in the ‘Materials and Methods’ section, total length was 530.5 Mb with an N50 length 751.5 kb. In the case of the assemblies using Canu and Wtdbg2, total lengths were 241.4 Mb and 390.1 Mb with N50 length 18.6 kb and 16.4 kb, and BUSCO completeness was 62.7% and 63.1%, whose genome coverages were low. The total length of Flye (527.5 Mb) was close to that of FALCON with parameter A (530.5 Mb), however, the N50 length of Flye (119.0 kb) was shorter than that of FALCON with parameter A (640.3 kb). In the case of NextDenovo, the total length of the primary sequence obtained by Purge_haplotigs was 372.4 Mb with the N50 length of 300.8 kb, which was close to the estimated genome size based on the *k*-mer frequency plot (402.0 Mb), however, the BUSCO completeness was 86.5%, indicating that the length of genome sequence might be longer than 372.4 Mb. According to these results, we concluded that the assembly result using FALCON parameter A is valid. Considering the assembly results using only Illumina short-reads, the key to the success of the genome assembly by long-reads in our study must be our establishment of a breeding system with artificial seawater, which allowed us to use 3 µg of genomic DNA from 1,500 clonal young medusae. The obtained genomic data will provide a valuable resource for further studies on rejuvenation, transdifferentiation, dedifferentiation, and ageing. Finally, although we here performed the GO enrichment analysis, it was difficult to reach any definitive conclusions in regard to the relationships between the characteristics of *T. dohrnii* and gene functions. Currently, we are trying to single-cell RNA-Seq and bulk RNA-Seq analyses to find clues to the mechanisms underlying rejuvenation.

## Supplementary Material

dsac047_suppl_Supplementary_Figures

dsac047_suppl_Supplementary_Table_S1-S9

dsac047_suppl_Supplementary_Table_S10
