# Supplementary material for: Genome assembly and transcriptomic analyses of the repeatedly rejuvenating jellyfish Turritopsis dohrnii
Source: DNA Res. 2022 Dec 15;30(1):dsac047. doi: 10.1093/dnares/dsac047 (PMC9835754; doi:10.1093/dnares/dsac047)
Supplement: dsac047_suppl_Supplementary_Figures [file dsac047_suppl_supplementary_figures.pdf]

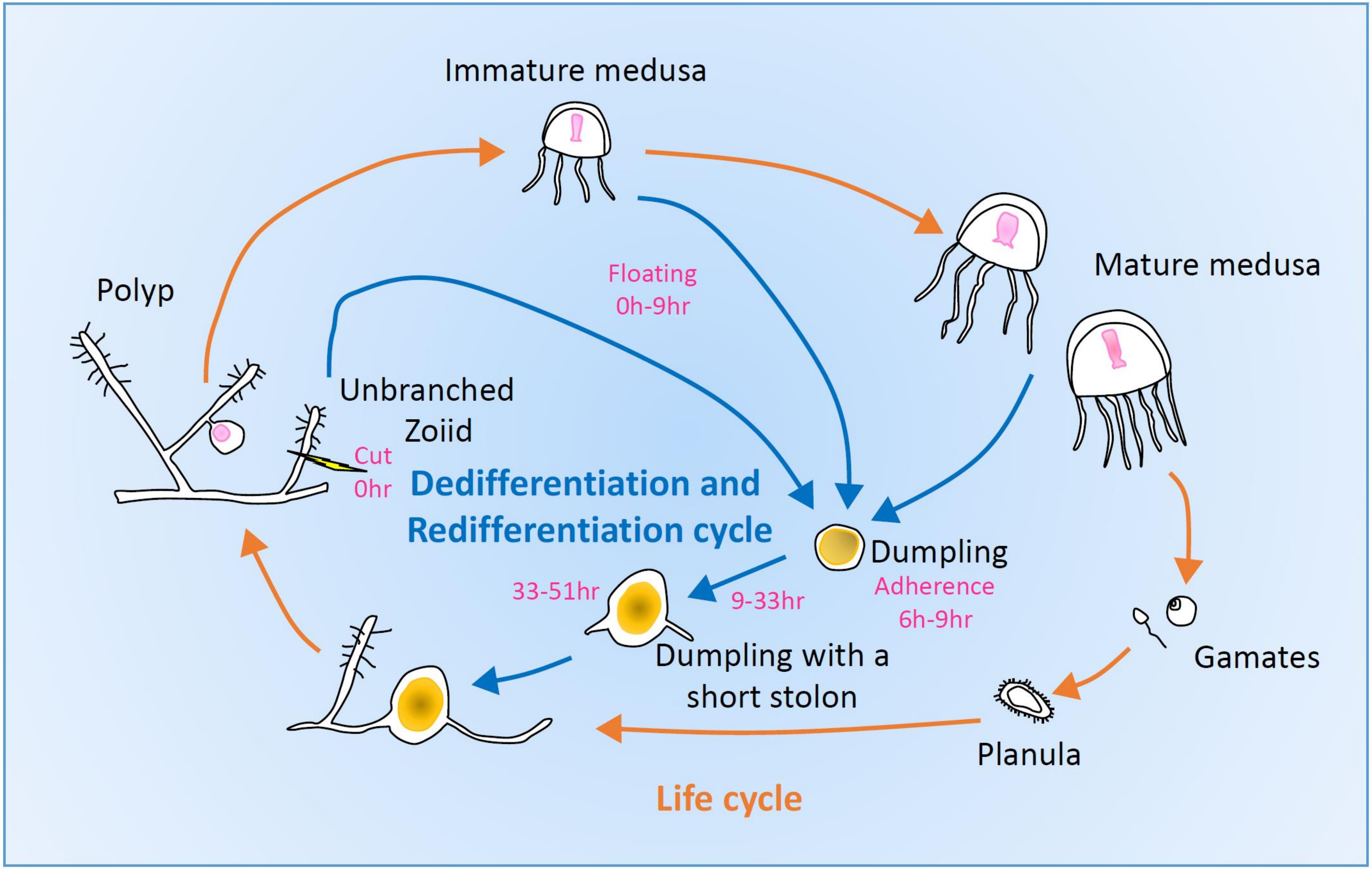

Supplementary Figure S1. Life cycle of *Turritopsis dohrnii*.
Orange arrows indicate the typical life cycle while blue indicates dedifferentiantion and redifferentiation cycle.

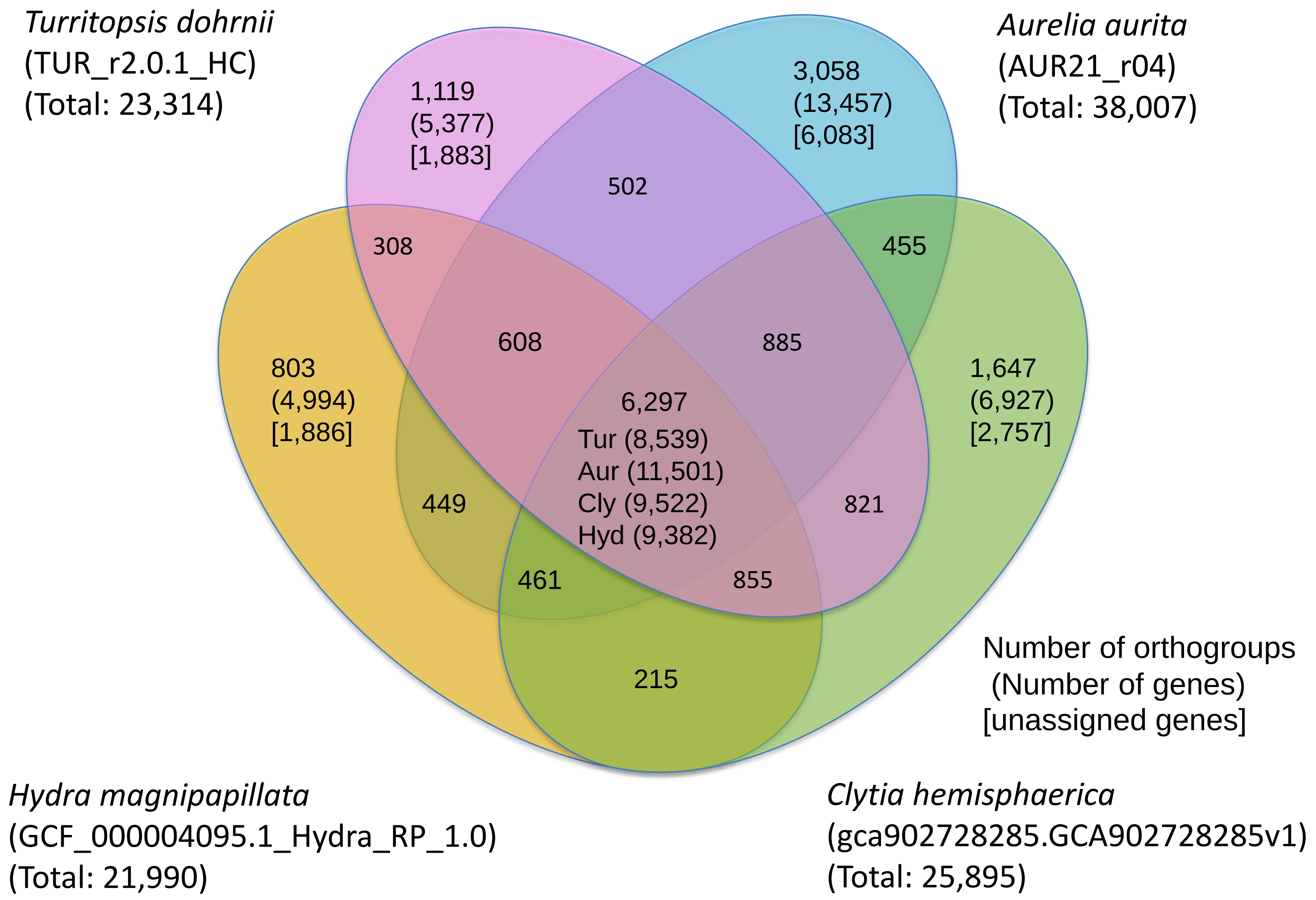

Supplementary Figure S2. Venn diagram of the four species
